# Four New Species and One New Record of *Thelephora* from China

**DOI:** 10.3390/jof10040300

**Published:** 2024-04-22

**Authors:** Mei-Zhi Tian, Hai-Bin Xia, Zheng-Lin Gao, Chang-Yin Zhao, Dan Ma, Zhu-Liang Yang, Yan-Chun Li

**Affiliations:** 1 Key Laboratory for Plant Diversity and Biogeography of East Asia, Kunming Institute of Botany, Chinese Academy of Sciences, Kunming 650201, China; tianmeizhi@mail.kib.ac.cn; 2 College of Life Sciences, Shanxi Normal University, Xi’an 710100, China; hbxia2001@163.com; 3 Yunnan Key Laboratory for Fungal Diversity and Green Development, Kunming 650201, China; 4 Amu Mountain Provincial Nature Reserve Management and Protection Bureau, Honghe 654400, China; gjlcjxny@163.com (Z.-L.G.); gao1378124@163.com (C.-Y.Z.); 13619626525@163.com (D.M.)

**Keywords:** Thelephorales, ectomycorhizal fungi, taxonomy, phylogeny, new taxa

## Abstract

Species of the genus *Thelephora* (Thelephorales, Thelephoraceae) are ectomycorrhizal symbionts of coniferous and broad-leaved plants, and some of them are well-known edible mushrooms, making it an exceptionally important group ecologically and economically. However, the diversity of the species from China has not been fully elucidated. In this study, we conducted a phylogenetic analysis based on the internal transcribed spacer (ITS) regions, using Maximum Likelihood and Bayesian analyses, along with morphological observations of this genus. Four new species from China are proposed, viz., *T. dactyliophora*, *T. lacunosa*, *T. petaloides*, and *T. pinnatifida*. In addition, *T. sikkimensis* originally described from India is reported for the first time from China. *Thelephora dactyliophora*, *T. pinnatifida*, and *T. sikkimensis* are distributed in subtropical forests and mainly associated with plants of the families Fagaceae and Pinaceae. *Thelephora lacunosa* and *T. petaloides* are distributed in tropical to subtropical forests. *Thelephora lacunosa* is mainly associated with plants of the families Fagaceae and Pinaceae, while *T. petaloides* is mainly associated with plants of the family Fagaceae. Line drawings of microstructures, color pictures of fresh basidiomes, and detailed descriptions of these five species are provided.

## 1. Introduction

*Thelephora* Ehrh. ex Willd. was originally described by Ehrhart and validly published by Willdenow with *T. terrestris* Ehrh. as its type species [1,2]. The genus is characterized by the diverse shapes of basidiomes which are stereoid, imbricate, rosette, infundibuliform, coralloid, or resupinate; the sulcate, zonate, glabrous to strigose, somewhat radially rugulose or wrinkled abhymenial surfaces; the smooth, slightly rugose to warty hymenial surfaces; the monomitic, clamped hyphal systems; the verruculose or echinulate basidiospore ornamentations; and the presence or absence of cystidia [1,2,3,4,5,6]. Species of the genus *Thelephora* are associated with a variety of plants of the families Pinaceae, Casuarinaceae, Ericaceae, Fagaceae, and Betulaceae. The mycelia of these species around the plant roots can help plants obtain essential minerals and water from the soil and resist diseases and drought [7], making significant contributions to plant health and ecosystem stability. In addition, some species in the genus play an essential role in the edible and medicinal fungal industry; e.g., *Thelephora aurantiotincta* Corner and *T. ganbajun* M. Zang have anticancer and anti-allergic effects [8,9,10]. Meanwhile, *T. ganbajun* is also one of the most popular edible mushrooms in China and some East Asian countries [11,12,13,14].

According to our preliminary statistics, 64 species of *Thelephora* have been accepted (http://www.indexfungorum.org/Names/Names.asp, accessed date: 25 February 2024), and they are mainly distributed in the northern temperate and tropical regions from Europe [15], North America [16,17,18,19,20], and Asia [21,22]. In China, 24 species have been reported [12,13,14,23,24,25]. During our recent research on *Thelephora* across China, we encountered five impressive species that have not been reported from this country. These species can be easily recognized by their conspicuous colors in the field. Molecular phylogenetic analyses of this genus based on the nuclear ribosomal internal transcribed spacer (ITS) indicated that they represent five distinct species. Combined with morphological characteristics, five species including four new to science: *T. dactyliophora* Yan C. Li & Zhu L. Yang, *T. lacunosa* Yan C. Li & Zhu L. Yang, *T. petaloides* Yan C. Li & Zhu L. Yang, and *T. pinnatifida* Yan C. Li & Zhu L. Yang, and one new to China, viz., *T. sikkimensis* K. Das, Hembrom & Kuha, were studied and documented herein.

## 2. Materials and Methods

### 2.1. Specimen Collections

Nineteen specimens of *Thelephora* were collected from China, including seventeen from southwestern China (Yunnan Province) and two from eastern China (Zhejiang Province). Photographs of these fresh specimens were taken in the field. Macroscopic characteristics and field notes including date, location, and habitat were made for each specimen, and then these specimens were dried using a fungal dryer and deposited in the fungal herbarium of the Herbarium KUN (Kunming Institute of Botany, Chinese Academy of Sciences) for taxonomic studies. Small pieces of each specimen were also dried in silica gel for molecular studies.

### 2.2. Morphological Studies

Macroscopic characteristics were obtained from specimen records and photos captured in the field. Color codes followed Kornerup and Wanscher [26,27]. Microscopic characteristics that include the structure of the pileipellis and the morphology of context hyphae, subhymenium hyphae, basidia, cystidia, and basidiospores were observed under a ZEISS Axiostar Plus microscope (Carl Zeiss AG, Oberkochen, Germany). Tissues were sectioned and mounted in 10% KOH, Cotton Blue (test for cyanophily), and Melzer’s reagent (test for amyloidity and dextrinoidity) [13,25,28,29]. Microscopic structures of pileipellis, hymenium, and subhymenium were drawn freehand; basidiospores and hyphae were measured at 1000× *g* magnification. The observations of basidiospore ornamentations were obtained with a ZEISS Sigma 300 scanning electron microscope (SEM) (Carl Zeiss AG, Oberkochen, Germany). For the measurement of basidiospores, at least 20 basidiospores were measured for each specimen; the notation “basidiospores (n/m/p)” is used to mean n basidiospores measured from m basidiomes of p specimens. L denotes spore length (arithmetic average of all spores); W denotes spore width (arithmetic average of all spores); Q denotes variation in the length/width ratios of basidiospores in side view; Q_m_ denotes the average Q of all basidiospores ± sample standard deviation [30,31,32]. Since the shape of basidiospore is mainly inferred by its length/width ratio, we have defined the terms of basidiospore shapes as follows: globose (length/width ratio = 1.01–1.05), subglobose to broadly ellipsoid (length/width ratio = 1.05–1.3), ellipsoid (length/width ratio = 1.3–1.6), and elongated (length/width ratio = 1.6–2). The abbreviation masl means meters above sea level [33]. 

### 2.3. DNA Extraction, PCR and DNA Sequencings

The extraction of genomic DNA from dried specimens was conducted using the CTAB method [34] and Ezup Column Fungi Genomic DNA Purification Kit (Shanghai Jinban Biotechnology Co., Ltd., Shanghai, China). PCR reactions contained 1 μL DNA solution (adjusted to approximately 20 ng), 1 µL of each primer, and 15 μL 2× Taq PCR Master Mix including Taq DNA Polymerase, MgCl_2_, and dNTP mix (Beijing Biomed Gene Technology Co., Ltd., Beijing, China). The final volume was adjusted to 30 μL with distilled sterile H_2_O. The amplification conditions were set as follows: denaturation at 95 °C for 4 min, 35 cycles of 30 s at 94 °C, 40 s at 53 °C, 1 min at 72 °C, a final extension of 8 min at 72 °C, and then coolant at 14 °C [35,36]. In this study, the internal transcribed spacer (ITS) regions were amplified with the primers ITS1F/ITS4 [30,34,37,38]. Some full lengths of ITS sequences that failed to be amplified were divided into two parts for amplification with primer pairs ITS1F/5.8S and 5.8SR/ITS4, respectively [39], and then sequences of these two parts were spliced together to obtain the full length of the ITS sequence. Sequences newly generated in this study were deposited in GenBank (Table 1).

### 2.4. Phylogenetic Analyses

DNA sequences were compiled with SeqMan (DNASTAR Lasergene 9). Sequences related to *Thelephora* downloaded from GenBank (https://www.ncbi.nlm.nih.gov/, accessed on 6 April 2024) and UNITE (https://unite.ut.ee/, accessed on 25 February 2024), and newly generated in this study were aligned with Mafft V7.490 [42] and manually adjusted with PhyDE when necessary. Then we performed phylogenetic analyses by Maximum Likelihood (ML) and Bayesian Inference (BI) approaches. The ML analysis was inferred with RaxmlGUI 2.0.10 [43] with model set as GTRGAMMAI for 1000 bootstrap replicates combined with an ML search. The BI analysis was conducted using MrBayes 3.2 [44] running 2,000,000 bootstrap replicates; the best-fit likelihood model employed for ITS dataset was HKY + I + G + F based on the Akaike Information Criterion (AIC) with ModelFinder [45,46]; the posterior probabilities (PP) were determined twice by running one cold and three heated chains in parallel mode, saving trees every 1000th generation. Other parameters were kept at their default settings. Runs were terminated once the average standard deviation of split frequencies went below 0.01. Genetic distances among ITS sequences were calculated using the Maximum Composite Likelihood model [47] in MEGA 11 [48].

## 3. Results

### 3.1. Phylogenetic Analyses

**Figure 1:**
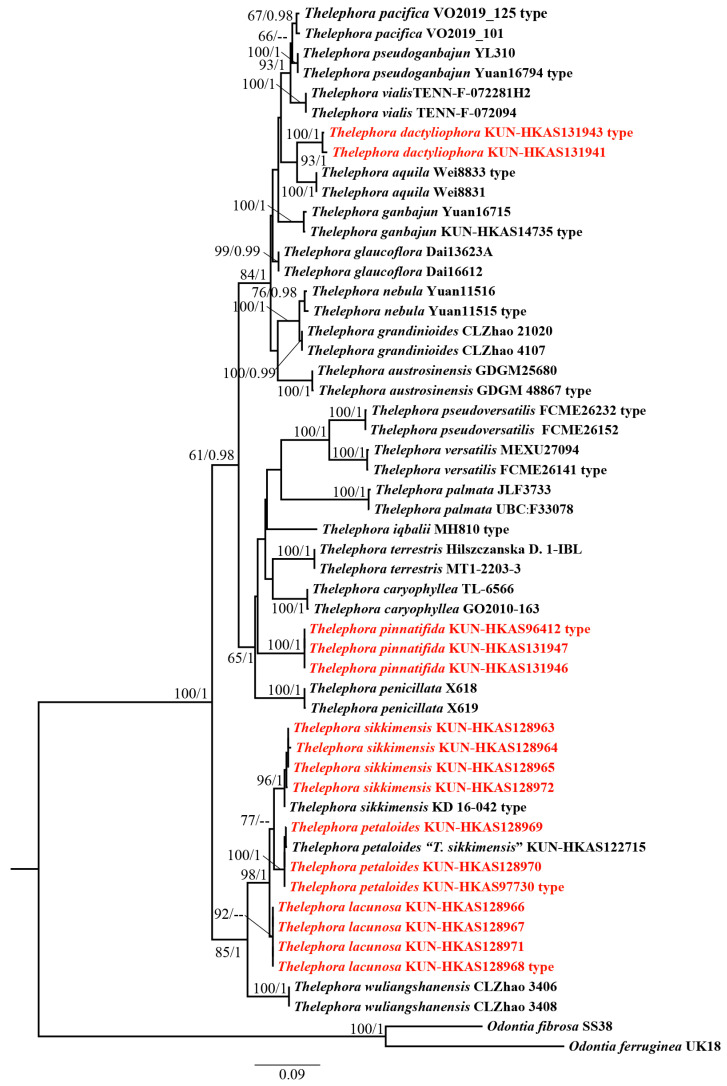
Phylogenetic relationships between species of *Thelelphora* inferred from ITS dataset using the Maximum Likelihood and Bayesian inference approaches (ML topology is shown). ML bootstrap support (≥50) and Bayesian posterior probability (≥0.95) are shown at the branches (BS/PP). Sequences newly generated in this study are highlighted in red. Vouchers are indicated after the species names. *Odontia fibrosa* and *O. ferruginea* were used as the outgroup taxa.

Phylogenetic analyses were conducted based on 53 ITS sequences of which 16 were newly generated in this study and 37 were downloaded from UNITE and GenBank (Table 1). *Odontia fibrosa* (Berk. & M.A. Curtis) Kõljalg and *O. ferruginea* Pers. were used as the outgroup taxa [14,18,22,47]. The alignment was submitted to TreeBASE (S30371). ML and BI approaches showed minimal differences in evaluation results; thus, only an ML tree was used for display (Figure 1). Our phylogenetic analyses indicated that *T. sikkimensis*, *T. petaloides*, and *T. lacunosa* cluster together with high support values (98/1). *T. dactyliophora* is sister to *T. aquila* S.R. Yang, Y.L. Wei & H.S. with moderate support values (93/1). *Thelephora pinnatifida* forms a separate branch with high support values (100/1). 

### 3.2. Analyses of Genetic Distances

In this study, the Maximum Composite Likelihood model was used to calculate the genetic distances within and between species of *Thelephora* used in this study. As shown in Table S1, the genetic distances of the ITS sequences within species ranged from 0 to 0.9%, with an average distance of 0.2%; the genetic distances between species ranged from 1.2% to 22.3%, with an average of 12.8%. 

### 3.3. Taxonomy

**Figure 2:**
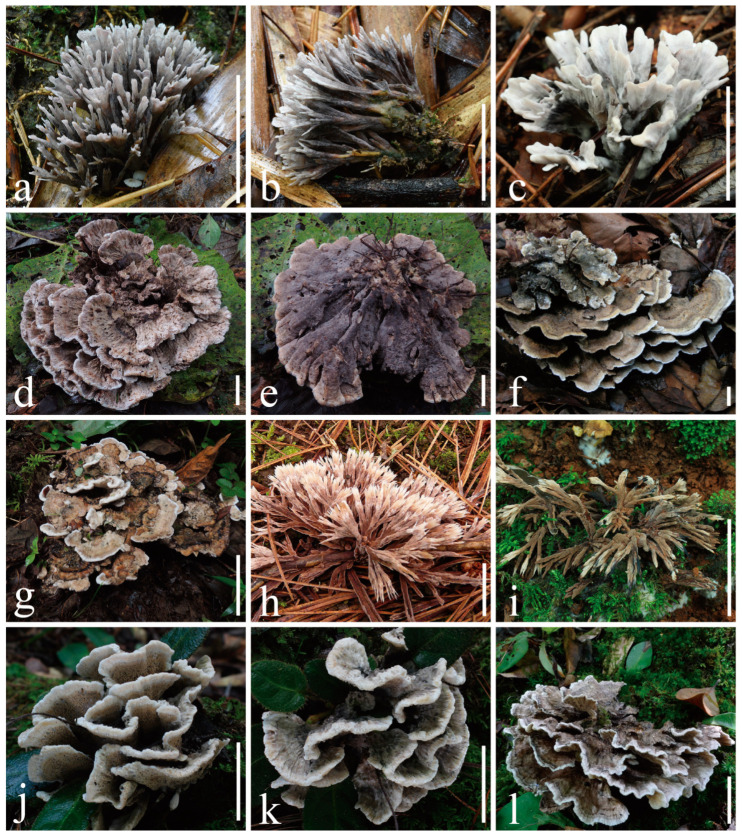
Fresh basidiomes. (**a**–**c**) *Thelephora dactyliophora* ((**a**,**b**) from KUN-HKAS131943, type; (**c**) from KUN-HKAS131941); (**d**,**e**) *Thelephora lacunosa* (KUN-HKAS128968, type); (**f**,**g**) *Thelephora petaloides* ((**f**) from KUN-HKAS97730, type; (**g**) from KUN-HKAS128969); (**h**,**i**) *Thelephora pinnatifida* ((**h**) from KUN-HKAS96412, type; (**i**) from KUN-HKAS131946); (**j**–**l**) *Thelephora sikkimensis* ((**j**,**k**) from KUN-HKAS128965, type; (**l**) from KUN-HKAS69236). Bars = 20 mm.

**Figure 3:**
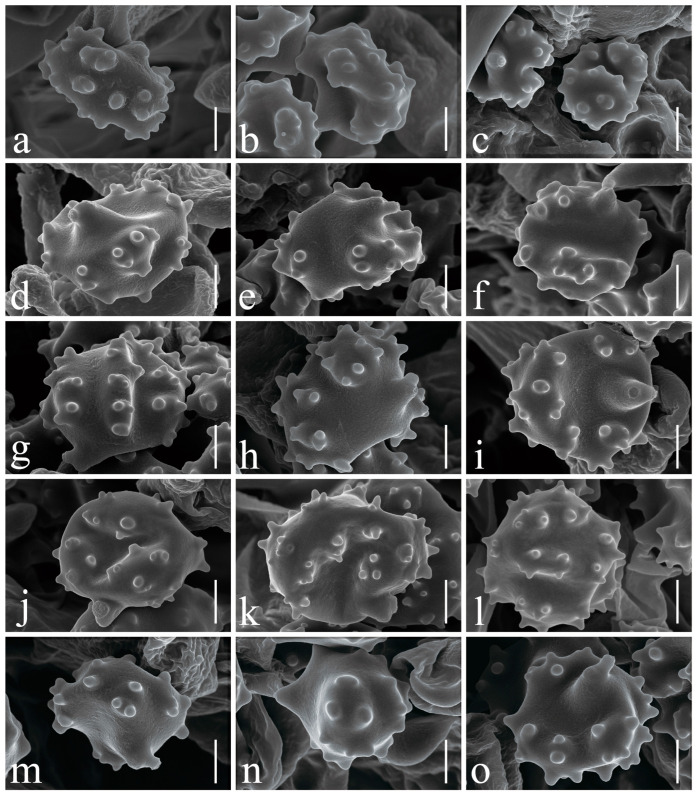
Basidiospores under SEM. (**a**–**c**) *Thelephora dactyliophora* (KUN-HKAS131943, type); (**d**–**f**) *Thelephora lacunosa* (KUN-HKAS128968, type); (**g**–**i**) *Thelephora petaloides* (KUN-HKAS97730, type); (**j**–**l**) *Thelephora pinnatifida* (KUN-HKAS96412, type); (**m**–**o**) *Thelephora sikkimensis* (KUN-HKAS128965, type). Bars = 2 µm.

**Figure 4:**
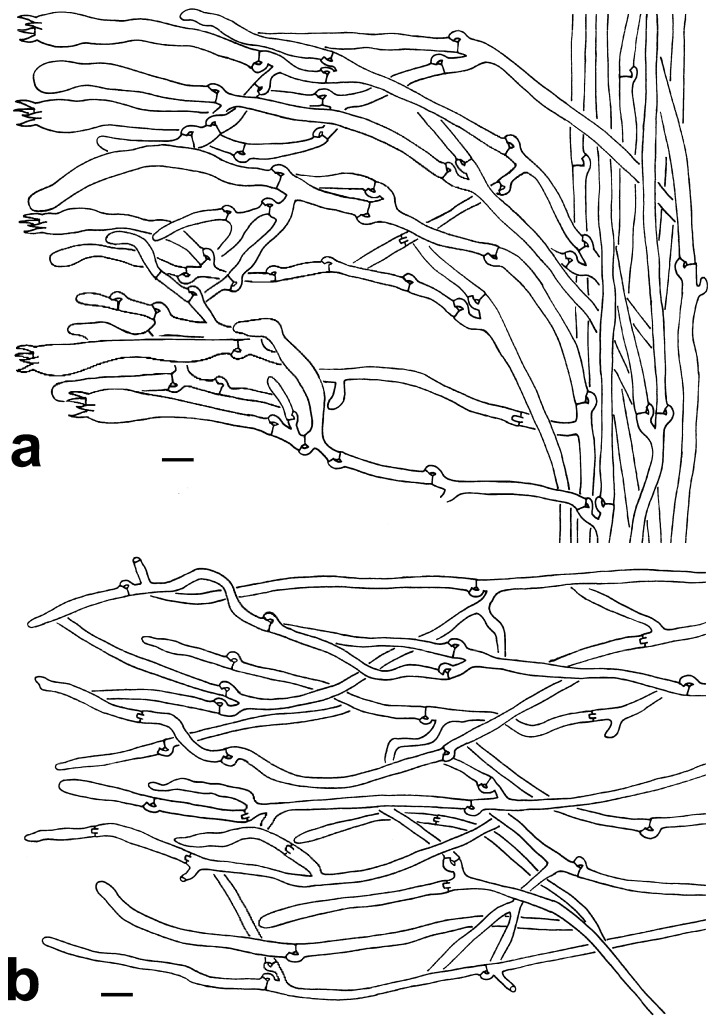
Microscopic features of *T. dactyliophora* (KUN-HKAS131943, type). (**a**) Hymenium and subhymenium. (**b**) Pileipellis. Bars = 10 µm.

***Thelephora dactyliophora*** Yan C. Li & Zhu L. Yang, sp. nov., Figure 2a–c, Figure 3a–c and Figure 4a,b.

MycoBank: 852625.

Etymology: dactyliophora refers to the finger-like branches of the basidiomes.

Diagnosis: Basidiomes coralloid; branch multiple ranks, finger-like to narrow flabelliform when young, spathulate to narrow petaloid when mature; abhymenial surface slightly rugulose, non-zonate, brownish gray to gray; hymenial surface concolorous with abhymenial surface, rugulose and non-zonate. Basidiospores nodulose to verrucose. This species is similar to *T. palmata* (Scop.) Fr. in its coralloid basidiomes, but differs in its slightly rugulose brownish gray to gray hymenial surface and relatively small basidiospores.

Type: China, Zhejiang Province: Lishui City, Jingning County, altitude 759 masl, 22 June 2021, Liu-Kun Jia 1577 (KUN-HKAS131943, GenBank Acc. No. OR940523).

Description: Basidiomes 35–65 mm high, 30–40 mm wide, gregarious to caespitose, humid and leathery when fresh, corky to hard and brittle when drying; branches arising from a shared stipe or center and arranged in coralloid shape; branch multiple ranks, flat, finger-like to narrow flabelliform when young, spathulate to narrow petaloid when mature; margin thin, about 0.1 mm thick, deeply lacerate. Abhymenial surface slightly rugulose, non-zonate, brownish gray to gray (6F2–6F1), but chalky white to orange-white (1A1–5A2) at margin. Hymenial surface concolorous with abhymenial surface, rugulose and non-zonate. Context 0.1–0.5 mm thick, relatively thin at margin and thick towards base, white (1A1), orange-white to gray-white (5A2–2B1), odor mild when fresh, sweet odor when drying. Stipe 10–20 × 2–5 mm, irregularly cylindrical to flatted or broadened at base, surface smooth to slightly rugose, brownish gray (6F2).

Basidia 45–72 × 6–9 µm, slightly clavate, hyaline, thin-walled, clamped at base, with four sterigmata; sterigmata 3.5–5.5 µm long and 1.5–2 µm wide at base, yellowish in Melzer’s reagent. Basidiospores (60/3/2) (4.5–) 5–7.5 × 4–6.5 µm (not including ornamentations), L = 6 µm, W = 5 µm, Q = 1–1.6, Q_m_ = 1.2 ± 0.15, subglobose to subellipsoid in side view, broadly ellipsoid to ellipsoid to irregularly lobed in ventral view, slightly thick-walled; surface nodulose to verrucose, ornamentation usually isolated, sometimes in groups from two to three, echinulis 0.3–0.5 µm high, subconical but apex somewhat obtuse; yellowish brown in 10% KOH, yellow to yellow-brown in Melzer’s reagent, non-amyloid, non-dextrinoid, cyanophilic. 

Monomitic hyphal system. Pileipellis: generative hyphae 3–5.5 µm wide, hyaline, subparallel to loosely interwoven, thin-walled, moderately branched, frequently branched near clamp connections, yellowish in Melzer’s reagent, non-amyloid, non-dextrinoid, acyanophilic. Context: generative hyphae 3.5–4.5 (–5.5) µm wide, hyaline, subparallel to loosely interwoven, thin-walled, moderately branched, frequently branched near clamp connections, yellowish in Melzer’s reagent, non-amyloid, non-dextrinoid, acyanophilic. Subhymenium: generative hyphae 3–5 µm wide, hyaline, subparallel to slightly divergent, thin-walled, frequently branched near clamp connections, yellowish in Melzer’s reagent, non-amyloid, non-dextrinoid, acyanophilic. 

Habitat: In subtropical evergreen broad-leaved forests dominated by plants of Fagaceae and Betulaceae, or in mixed forests dominated by plants of Fagaceae and Pinaceae.

Distribution: Currently known from Zhejiang Province (eastern China).

Additional specimens examined: China, Zhejiang Province: Lishui, Jingning County, Wangdongyang Forest Wetland National Park, 1321 masl, 11 September 2021, Geng-Shen Wang 1795 (KUN-HKAS131941); the same location, 15 July 2022, Peng-Cheng Yuan 698 (KUN-HKAS131940).

Notes: *Thelephora dactyliophora* is phylogenetically related to *T. aquila*. These two species share similar sizes of basidiomes (50 × 40 mm in *T. aquila* vs. 35–65 × 30–40 mm in *T. dactyliophora*) and similar sizes of basidiospores (5−7.3 × 4−6.5 µm in *T. aquila* vs. 4.5–7.5 × 4–6.5 µm in *T. dactyliophora*). However, *T. aquila* is easily distinguished from *T. dactyliophora* by its flabelliform to applanate–lobate branches of basidiomes and its black and zonate abhymenial surface which is somewhat radially rugulose or wrinkled [25]. The coralloid basidiomes of *T*. *dactyliophora* are very similar to those of *T. palmata*. However, *T. palmata* is clearly different from *T*. *dactyliophora* in its velvet brownish purple to black or purple-brown branches and relatively large basidiospores (7.4–9.3 × 8–11.1 µm in *T. palmata* vs. 4.5–7.5 × 4–6.5 µm in *T. dactyliophora*) [20,49].

**Figure 5:**
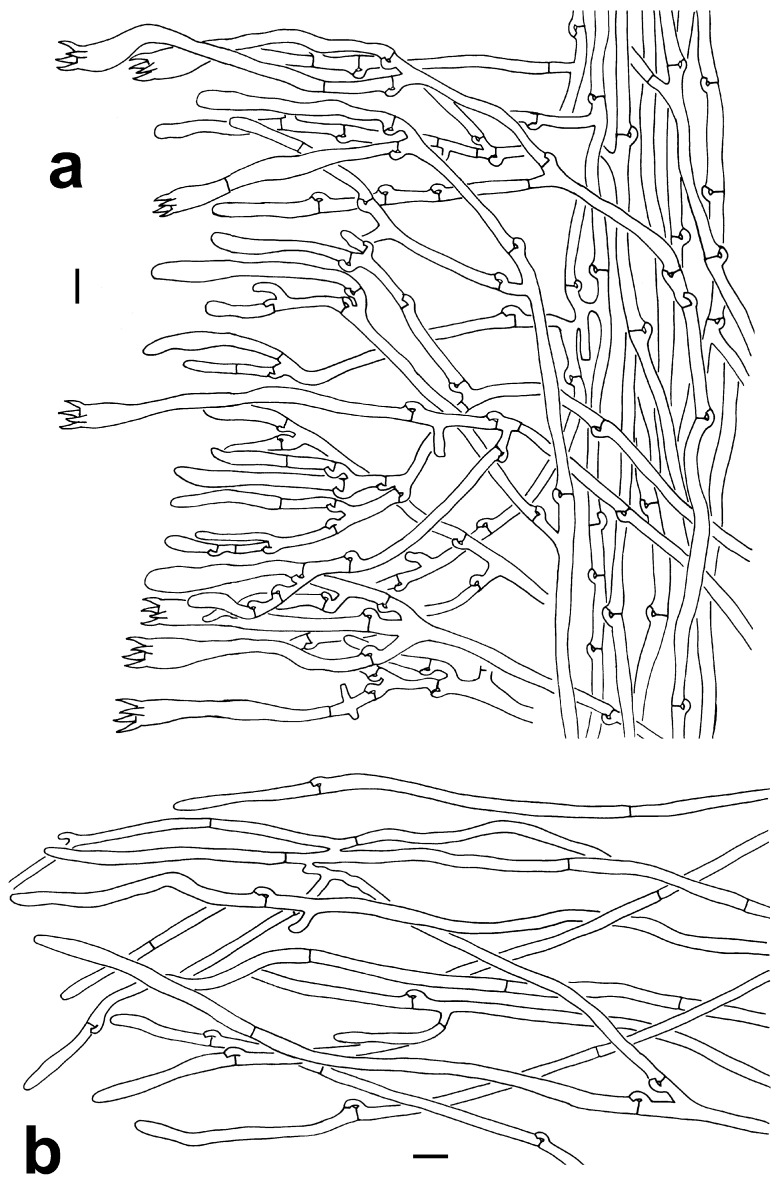
Microscopic features of *T*. *lacunosa* (KUN-HKAS128968, type). (**a**) Hymenium and subhymenium. (**b**) Pileipellis. Bars = 10 µm. Bars = 2 µm.

***Thelephora lacunosa*** Yan C. Li & Zhu L. Yang, sp. nov., Figure 2d,e, Figure 3d–f and Figure 5a,b.

MycoBank: 852626.

Etymology: lacunosa refers to the lacunary abhymenial surface.

Diagnosis: Basidiomes imbricate, rosette to flabelliform; branches flabelliform, petaloid to applanate–lobate; abhymenial surface lacunary, sulcate, irregularly ridged, non-zonate, and warty, brown to grayish brown or grayish orange at center then gradually becoming paler towards margin; hymenial surface imperceptibly rugose and warty, brownish orange to gray or purple-gray at center and becoming pale orange towards margin. Basidiospores nodulose to verrucose. This species is morphologically similar to *T. ganbajun* in the imbricate to rosette basidiomes, but it differs in its lacunary, irregularly ridged, non-zonate, brown to grayish brown or grayish orange abhymenial surface and brownish red stipe.

Type: China, Yunnan Province: Honghe, Jiache Town, 2140 masl, 22 September 2022, Yan-Chun Li 3913 (KUN-HKAS128968, GenBank Acc. No. OR512335).

Description: Basidiomes 67–80 mm high, 80–150 mm wide, solitary, gregarious to caespitose, humid and leathery when fresh, corky to hard and brittle when drying; branches arising from a shared stipe or center and arranged in imbricate, rosette to flabelliform; branches flat, flabelliform, petaloid to applanate–lobate; margin 0.5–3 mm thick, nearly entire, rarely lacerate, imperceptibly wavy. Abhymenial surface lacunary, sulcate, irregularly ridged, non-zonate, and warty, brown to grayish brown (6E5–7E3) or grayish orange (6B3) at center then gradually becoming paler towards margin, but white to pale orange-gray (5A1–5B2) at margin. Hymenial surface imperceptibly rugose and warty, brownish orange to gray (6B4–7D2) or purple-gray (14E2) at center and becoming pale orange (6A3) towards margin, but orange-white (5A2) at margin. Context 0.5–3.5 mm thick, yellowish white to gray-white (2A2–2B1), odor mild when fresh, yeast powder flavor when drying. Stipe 20–25 × 12–35 mm, subconical to broadened or flatted towards base, surface rugose, glabrous, brownish red to brownish yellow (7E8–5D8).

Basidia 72–100 × 8–12 µm, subclavate, hyaline, thin-walled, clamped at base, with four sterigmata; sterigmata 3–6 µm long and 1.5–3 µm wide at base, yellowish in Melzer’s reagent. Basidiospores (100/5/4) 4.5–6.5 × 4–5.5 µm (not including ornamentations), L = 5.5 µm, W = 5 µm, Q = 1–1.5, Q_m_ = 1.1 ± 0.11, subglobose to subellipsoid in side view, broadly ellipsoid to ellipsoid to irregularly lobed in ventral view, slightly thick-walled, pale yellowish brown in 10% KOH, yellow-brown to brown in Melzer’s reagent, non-amyloid, non-dextrinoid, cyanophilic; surface nodulose to verrucose, ornamentation usually isolated, sometimes in groups from two to three, echinulis 0.4–0.5 µm high, subconical but somewhat obtuse at apex. 

Monomitic hyphal system. Pileipellis: generative hyphae 5–6 µm wide, hyaline to yellowish, subparallel to loosely interwoven, thin-walled, frequently branched near clamp connections, commonly with simple septa, yellowish in Melzer’s reagent, non-amyloid, non-dextrinoid, acyanophilic. Context: generative hyphae 3–5.5 µm wide, hyaline, subparallel to loosely interwoven, thin-walled, frequently branched near the clamp connections, with a few simple septa, yellowish in Melzer’s reagent, non-amyloid, non-dextrinoid, acyanophilic. Subhymenium: generative hyphae 2.5–4.5 µm wide, hyaline, subparallel to slightly divergent, thin-walled, moderately branched, with clamp connections and a few simple septa, yellowish in Melzer’s reagent, non-amyloid, non-dextrinoid, acyanophilic.

Habitat: In subtropical evergreen broad-leaved forests dominated by plants of Fagaceae, or in subtropical mixed forests dominated by plants of Fagaceae and Pinaceae.

Distribution: Currently known from Yunnan Province (southwestern China).

Additional specimens examined: China, Yunnan Province: Honghe, Jiache Town, Sanjian Mountains, 2140 masl, 22 September 2022, Yan-Chun Li 3971 (KUN-HKAS128971); Puer, Taiyanghe Nature Reserve, 1400 masl, 2 July 2022, Yan-Chun Li 3307 (KUN-HKAS128966); the same date and location, Yan-Chun Li 3311 (KUN-HKAS128967).

Notes: In morphology, *T. lacunosa* shares similar features with *T. sikkimensis*: the flabelliform to petaloid branches of basidiomes with thin and imperceptibly wavy margin; the warty and non-zonate hymenial surface; and the imbricate, rosette basidiomes [22]. However, *T. lacunosa* differs from *T. sikkimensis* clearly in the lacunary, non-zonate, and grayish brown to grayish orange abhymenial surface and the subconical stipe. The yeast powder flavor when drying, the imbricate to rosette basidiomes, and the flabelliform branches of basidiomes make *T. ganbajun* similar to *T. lacunosa*, but *T. ganbajun* has an inconspicuously gray to black abhymenial surface and a brown to yellowish brown stipe which is cylindrical to flatted or broadened [25]. *Thelephora lacunosa* and *T. pseudoganbajun* S.R. Yang & H.S. Yuan share some common features including the imperceptibly wavy margin of the branches and the imbricate to rosette basidiomes [25]. However, *T. pseudoganbajun* differs from *T. lacunosa* in its brown to yellowish brown and somewhat radially rugulose or wrinkled abhymenial surface which is obscurely zonate, radially rugulose or longitudinally wrinkled hymenial surface, relatively small basidia (45–65 × 6–10 µm in *T. pseudoganbajun* vs. 72–100 × 8–12 µm in *T. lacunosa*), and occasionally isotypical clamp connections symmetrically growing on both sides of the hyphae [25]. *Thelephora pacifica* is similar to *T. lacunosa* due to its grayish brown abhymenial surface and similar size of basidiospores (4.3–7.3 × 3.6–6 µm in *T. pacifica* vs. 4.5–6.5 × 4–5.5 µm in *T. lacunose*), but *T. pacifica* differs from *T. lacunosa* in its pale olive-yellow margin of basidiomes, dark grayish brown stipe, and relatively small basidia (19.5–30 × 4.7–6.3 µm in *T. pacifica* vs. 72–100 × 8–12 µm in *T. lacunosa*) [41].

**Figure 6:**
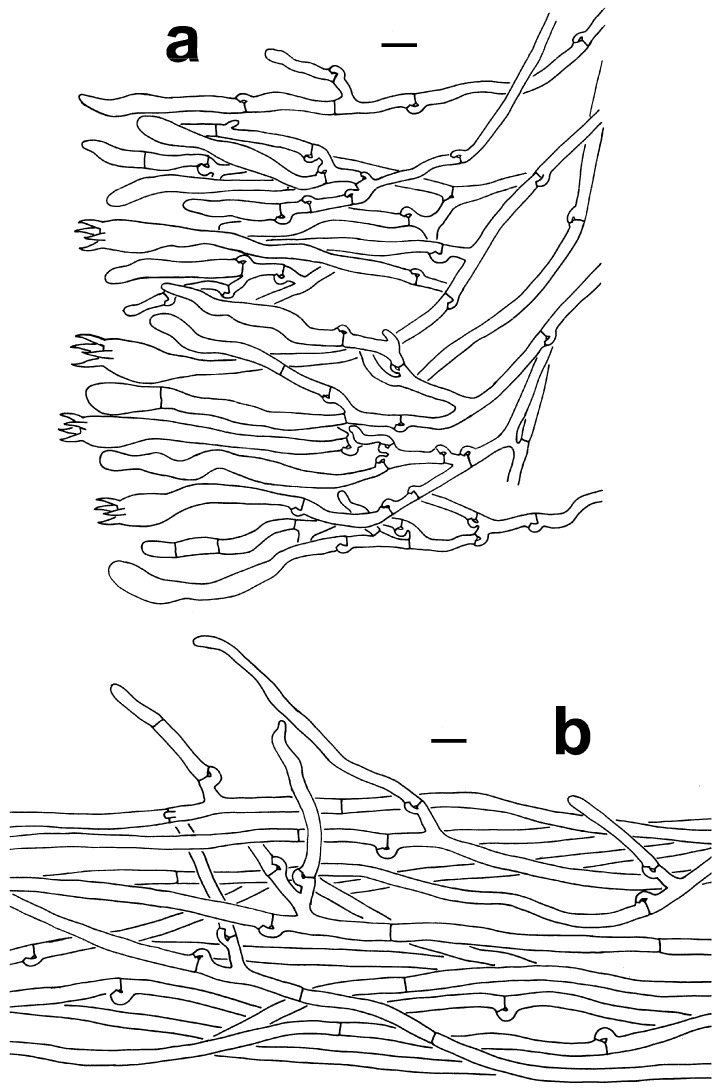
Microscopic features of *T. petaloides* (KUN-HKAS97730, type). (**a**) Hymenium and subhymenium (KUN-HKAS97730, type). (**b**) Pileipellis. Bars = 10 µm.

***Thelephora petaloides*** Yan C. Li & Zhu L. Yang, sp. nov., Figure 2f,g, Figure 3g–i and Figure 6a,b.

MycoBank: 852627.

Etymology: petaloides refers to the petaloid basidiomes.

Diagnosis: Basidiomes imbricate to umbrella; branches flabelliform to applanate–lobate; abhymenial surface sulcate, somewhat irregularly ridged, glabrous, zonate, orange-yellow to grayish orange or pale orange elsewhere; hymenial surface imperceptibly rugose, warty, zonate near margin, bright orange elsewhere. Basidiospores nodulose to verrucose. This species is similar to *T. sikkimensis* in its sulcate abhymenial surface and warty hymenial surface but differs in its orange-gray and densely warty hymenial surface and visibly wavy margin of basidiomes.

Type: China, Yunnan Province: Puer City, Lancang County, Huimin Town, 1410 masl, 26 September 2016, Jian-Wei Liu 530828MF-226 (KUN-HKAS97730, GenBank Acc. No. OR512334). 

Description: Basidiomes (40–) 80–100 mm high, 55–140 (–220) mm wide, solitary to concrescent, humid and leathery when fresh, corky to hard and brittle when drying; branches arising from a shared stipe or center and arranged in imbricate to umbrella shape; branches flat, flabelliform, applanate–lobate; margin 1–2 mm thick, nearly entire, rarely lacerate, visibly wavy. Abhymenial surface sulcate, somewhat irregularly ridged, glabrous, zonate, brownish gray (6E2) at center, orange-yellow to grayish orange (6A8–6B3) or pale orange (5A2) elsewhere, but chalky white (1A1) at margin. Hymenial surface imperceptibly rugose, warty, zonate near margin, orange-gray (5B2) at center, becoming bright orange (5A4) elsewhere, but chalky white (1A1) at margin. Context 1–4 mm thick, relatively thin at margin and thick towards center, white to yellowish white (1A1–2A2), odor mild when fresh, yeast powder flavor when drying. Stipe 10–40 × 9–13 mm, irregularly cylindrical to flatted or broadened at base, surface rugose, glabrous, brownish gray (5E2).

Basidia 53–92 × 9–12 µm, subclavate, pale yellow to pale yellowish brown, thin-walled, clamped at base, with four sterigmata; sterigmata 3–5 µm long and 2.5–3 µm wide at base, yellowish in Melzer’s reagent. Basidiospores (100/5/5) 4.5–7.5 × 3.5–6.5 µm (not including ornamentations), L = 6 µm, W = 5.2 µm, Q = 1–1.71, Q_m_ = 1.16 ± 0.14, subglobose to subellipsoid or sometimes elongated in side view, globose to ellipsoid to irregularly lobed in ventral view; surface nodulose to verrucose, slightly thick-walled; ornamentation in groups from one to two, echinulis 0.5–0.7 µm high, subconical but apex somewhat obtuse; pale yellowish brown in 10% KOH, yellow-brown to brownish in Melzer’s reagent, non-amyloid, non-dextrinoid, cyanophilic. 

Monomitic hyphal system. Pileipellis: generative hyphae 4–5 µm wide, hyaline, subparallel to loosely interwoven, thin-walled, moderately branched, with clamp connections and a few simple septa, yellowish in Melzer’s reagent, non-amyloid, non-dextrinoid, acyanophilic. Context: generative hyphae 4–5 µm wide, hyaline, subparallel to loosely interwoven, thin-walled, moderately branched, with clamp connections and a few simple septa, yellowish in Melzer’s reagent, non-amyloid, non-dextrinoid, acyanophilic. Subhymenium: generative hyphae 2–4 µm wide, pale yellow to pale yellowish brown, subparallel to slightly divergent, thin-walled, frequently branched near clamp connections, with a few simple septa, yellowish in Melzer’s reagent, non-amyloid, non-dextrinoid, acyanophilic.

Habitat: In subtropical forests dominated by plants of Fagaceae.

Distribution: Currently known from Yunnan Province (southwestern China).

Additional specimens examined: China, Yunnan Province: Honghe, Jiache Town, Sanjian Mountains, 2140 masl, 22 September 2022, Yan-Chun Li 3918 (KUN-HKAS128969); the same date and location, Yan-Chun Li 3960 (KUN-HKAS128970); Baoshan, Longling County, 1700 masl, 12 September 2002, Zhu L. Yang 3581 (KUN-HKAS42087); Nujiang, Lushui County, Pianma Town, 1850 masl, 1 April 2001, Fu-Qiang Yu 696 (KUN-HKAS38935).

Notes: In our phylogenetic analyses, *T. petaloides*, *T. sikkimensis*, *T. lacunosa*, and *T. wuliangshanensis* clustered together with high support values (85/1) (Figure 1). However, *T. wuliangshanensis* is characterized by its infundibuliform basidiomes, the buff to salmon abhymenial surface with radially black striations, and the umber to coffee and nearly glabrous hymenial surface [14]. Morphologically, *T. petaloides* and *T. lacunosa* share similar characteristics in the flat, flabelliform branches of basidiomes and warty hymenial surface. However, *T. lacunosa* can be distinguished by its imperceptibly wavy margin of basidiomes; lacunary, sulcate, irregularly ridged, non-zonate, and warty abhymenial surface which is brown, grayish brown to grayish orange but white to pale orange-gray at the margin; and brownish orange hymenial surface with orange-white tint at the margin. In addition, the sulcate abhymenial surface, the flat and flabelliform branches of basidiomes, the white context, and the warty hymenial surface make *T. petaloides* similar to *T. sikkimensis*. However, *T. sikkimensis* is obviously distinguished from *T. petaloides* in its relatively small basidiomes (45–67 × 50–70 mm in *T. sikkimensis* vs. 40–100 × 55–220 mm in *T. petaloides*); grayish black to purplish gray or grayish green abhymenial surface which is chalky white to orange-white at margin; smooth to irregularly plicate hymenium surface which is pale orange to purplish gray or grayish green, but orange-white to almost white at the margin; and rugose and warty stipe which is pale orange or sometimes purplish gray.

**Figure 7:**
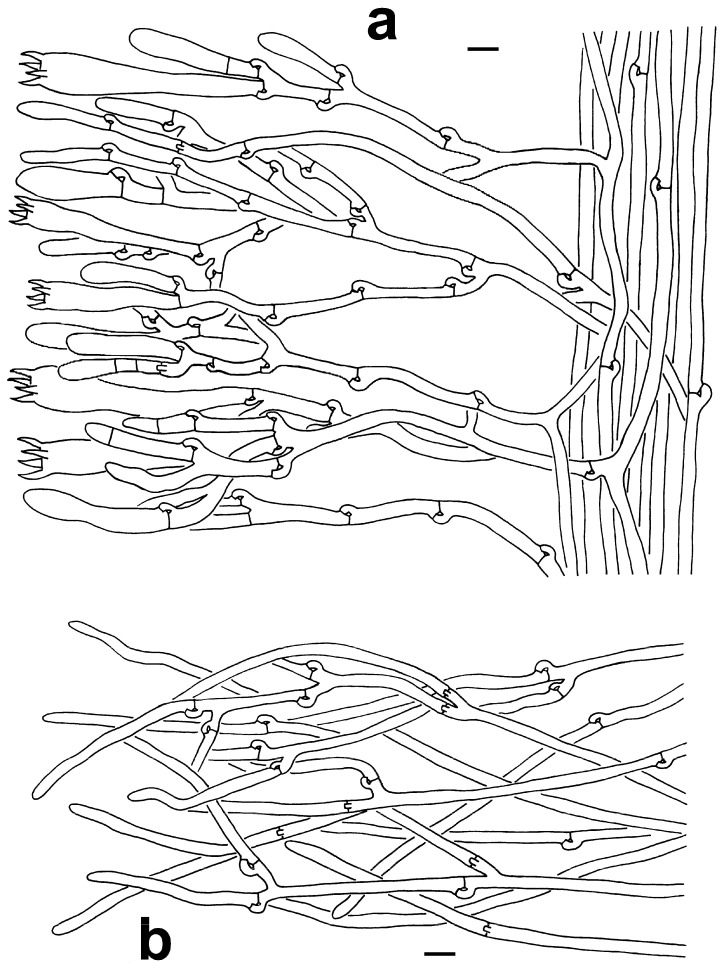
Microscopic features of *T*. *pinnatifida* (KUN-HKAS96412, type). (**a**) Hymenium and subhymenium. (**b**) Hair-like appendages of pointed tips. Bars = 10 µm.

***Thelephora pinnatifida*** Yan C. Li & Zhu L. Yang, sp. nov., Figure 2h,i, Figure 3j–l and Figure 7a,b.

MycoBank: 852628.

Etymology: pinnatifida refers to the pinnatifid margin of branches of the basidiomes.

Diagnosis: Basidiomes clavarioid; branch clavate to pinnatifid or ramiform with tips needle-like; surface sulcate, non-zonate, visibly ribbed, brownish orange to brown. Basidiospores nodulose to verrucose. This species is similar to *T. iqbalii* Khalid & Hanif in its clavarioid basidiomes and needle-like tips of branches but differs in its brownish orange to brown basidiomes with chalky white to orange-white margin and relatively long basidia.

Type: China, Yunnan Province: Kunming, Chongming County, Dian Yuan Town, Liangwang Mountain, Jiaochangba, 2622 masl, 23 July 2016, Xiang-Hua Wang 3823 (KUN-HKAS96412, GenBank Acc. No. OR940526).

Description: Basidiomes 35–45 mm high, 28–85 mm wide, gregarious to caespitose, humid and leathery when fresh, corky when drying; branches arising from a shared stipe or center and arranged in clavarioid shape, sometimes sub-resupinate to resupinate; branch clavate to pinnatifid or ramiform with tips needle-like, surface sulcate, non-zonate, visibly ribbed, brownish orange to brown (5C5–7E8), but chalky white to orange-white (1A1–5A2) at margin; margin 0.1–0.5 mm thick, deeply lacerate, velvety. Context 0.1–2 mm thick, relatively thin at margin and thick towards center, yellowish white to brown (2A2–7E8), odor mild when fresh, yeast powder flavor when drying. Stipe (0–) 10–15 × (0–) 3–10 mm, irregularly cylindrical, surface rugose, brown (7E7).

Hymenium predominantly amphigenous. Basidia 47–90 × 10–11.5 µm, nearly clavate, pale yellow, thin-walled, clamped at base, with four sterigmata; sterigmata 5–8 (–10) µm long and 1.5–3 (–4) µm wide at base, yellowish in Melzer’s reagent. Basidiospores (80/4/4) (5.5–) 6–9.5 × 5–8.5 µm (not including ornamentations), L = 7.6 µm, W = 6.4 µm, Q = 1–1.7, Q_m_ = 1.19 ± 0.15, subglobose to subellipsoid or sometimes elongated in side view, globose to broadly ellipsoid or ellipsoid to irregularly lobed in ventral view, slightly thick-walled; surface verrucose to subechinate, ornamentation usually isolated, sometimes in groups of two or more, echinulis 0.3–0.5 (–0.6) µm high, subconical but apex somewhat obtuse; pale yellowish brown in 10% KOH, yellow-brown to brown in Melzer’s reagent, non-amyloid, non-dextrinoid, cyanophilic. 

Monomitic hyphal system. Hair-like appendages of pointed tips: generative hyphae 3.5–5 (–5.5) µm wide, subparallel to loosely interwoven, thin-walled, often branched near clamp connections, hyaline in 10% KOH, yellow to pale yellow in Melzer’s reagent, non-amyloid, non-dextrinoid, acyanophilic. Context: generative hyphae 4.5–6.5 µm wide, pale yellow, subparallel to loosely interwoven, thin-walled, moderately branched, frequently branched often near clamp connections, septate absent, yellowish in Melzer’s reagent, non-amyloid, non-dextrinoid, acyanophilic. Subhymenium: generative hyphae 4–5.5 µm wide, pale yellow, subparallel to slightly divergent, thin-walled, moderately branched, frequently branched often near clamp connections, septate absent, yellowish in Melzer’s reagent, with acyanophilic reaction in Cotton Blue.

Habitat: In subtropical broad-leaved forests dominated by plants of Fagaceae, or in coniferous forests dominated by plants of Pinaceae.

Distribution: Currently known from Yunnan Province (southwestern China).

Additional specimens examined: China, Yunnan Province: Kunming, Chongming County, Dianyuan Town, Liangwang Mountain, Jiaochangba, 2622 masl, 23 July 2016, Xiang-Hua Wang 3823 (KUN-HKAS96412); Kunming, Kunming Botanical Garden, 1977 masl, 15 August 2023, Mei-Zhi Tian 394 (KUN-HKAS131946) and Mei-Zhi Tian 395 (KUN-HKAS131947); Dali, Jianchuan County, Diannan Town, Fada Village, 2200 masl, 10 August 2023, Mei-Zhi Tian 392 (KUN-HKAS131944) and Mei-Zhi Tian 393 (KUN-HKAS131945).

Notes: *Thelephora pinnatifida* can be confused with *T. iqbalii*, *T. penicillata* (Pers.) Fr., *T. pseudoversatilis* Ram.-Lóp. & Villegas, and *T. versatilis* Ramírez-López & M. Villegas due to their clavarioid basidiomes [4,18,41]. However, they cluster into five independent lineages and represent five distinct species in our phylogenetic analyses. Furthermore, *T. iqbalii* differs from *T*. *pinnatifida* in its grayish brown basidiomes with pinkish white margin of branches and relatively short basidia (40–55 × 6–10 µm in *T. iqbalii* vs. 47–90 × 10–11.5 µm in *T. pinnatifida*) [41]. *Thelephora penicillata* differs from *T. pinnatifida* in its fuscous purple basidiomes and much higher echinulis on the surface of basidiospores (echinulis up to 1.5 µm high in *T. penicillata* vs. 0.3–0.6 µm high in *T. pinnatifida*) [4]. *Thelephora pseudoversatilis* differs from *T. pinnatifida* in its much higher echinulis on the surface of basidiospores (echinulis 1–2 µm high in *T. pseudoversatilis* vs. 0.3–0.6 µm high in *T. pinnatifida*). *Thelephora versatilis* differs from *T. pinnatifida* in its clavate branches without pinnatifid margin and much higher echinulis on the surface of basidiospores (echinulis 0.5–1.5 high in *T. versatilis* vs. 0.3–0.6 µm high in *T. pinnatifida*). 

**Figure 8:**
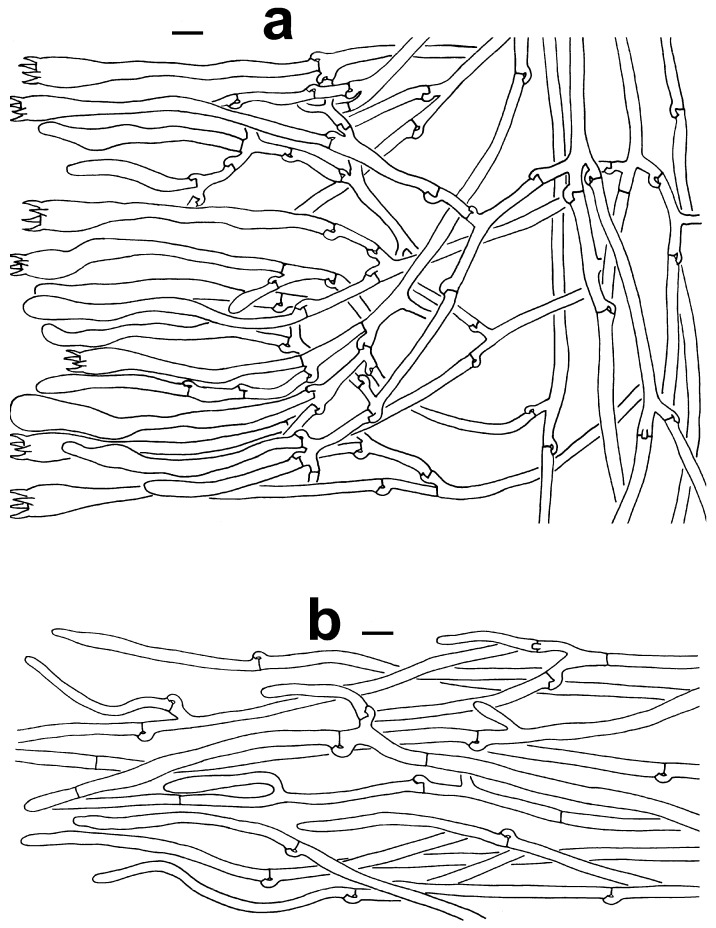
Microscopic features of *T. sikkimensis* (KUN-HKAS128965, type). (**a**) Hymenium and subhymenium. (**b**) Pileipellis. Bars = 10 µm.

***Thelephora sikkimensis*** K. Das, Hembrom & Kuha, Figure 2j–l, Figure 3m–o and Figure 8a,b.

Description: Basidiomes 45–67 mm high, 50–70 mm wide, gregarious to caespitose, humid and leathery when fresh, corky to hard and brittle when drying, branches arising from a shared stipe or center and arranged in imbricate to rosette shape; branches effused to reflexed, flabelliform to petaloid, margin thin about 1 mm thick, nearly entire, rarely lacerate, imperceptibly wavy. Abhymenial surface sulcate, indistinctly zonate, radially rugulose or wrinkled, grayish black (18F1) at center, purplish gray to grayish green (13E2–25E5) elsewhere, but chalky white to orange-white (1A1–5A2) at margin. Hymenial surface rugose, warty, gradually from smooth to irregularly plicate near the margin, non-zonate, pale orange (5A4) when young, purplish gray (13E2) to grayish green (24C1) when mature, but orange-white to almost white (5A2–1A1) at margin. Context 1–2 mm thick, relatively thin at margin and thick towards center, white, odor mild when fresh, yeast powder flavor when drying. Stipe 12–14 × 2–9 mm, irregularly cylindrical, surface rugose and warty, pale orange (5A3), sometimes purplish gray (13E2).

Basidia 72–100 × 7.5–10 µm, subclavate to subcylindrical, hyaline, thin-walled, clamped at base, with four sterigmata; sterigmata 4–6 µm long and 2–3 µm wide at base, yellowish in Melzer’s reagent. Basidiospores (80/4/4) 4.5–6 (–6.5) × 4–5.5 µm (not including ornamentations), L = 5.6 µm, W = 5 µm, Q = 1–1.38, Q_m_ = 1.13 ± 0.09, subglobose to subellipsoid in side view, globose to broadly ellipsoid or sometimes ellipsoid to irregularly lobed in ventral view, slightly thick-walled; surface nodulose to verrucose, ornamentation in groups from one to two, echinulis 0.3–0.7 µm high, subconical but apex somewhat obtuse; pale brown in 10% KOH, yellowish brown to brownish in Melzer’s reagent, non-amyloid, non-dextrinoid, cyanophilic.

Monomitic hyphal system. Pileipellis: generative hyphae 3–5 µm wide, hyaline, subparallel to loosely interwoven, thin-walled, moderately branched, with clamp connections and a few simple septa, yellowish in Melzer’s reagent, with acyanophilic reaction in Cotton Blue. Context: generative hyphae 2.5–5.5 µm wide, hyaline, subparallel to loosely interwoven, thin-walled, frequently branched near clamp connections, with a few simple septa, yellowish in Melzer’s reagent, with acyanophilic reaction in Cotton Blue. Subhymenium: generative hyphae 2–4 µm wide, hyaline, subparallel to slightly divergent, thin-walled, frequently branched near clamp connections, with a few simple septa, yellowish in Melzer’s reagent, with acyanophilic reaction in Cotton Blue.

Habitat: In subtropical mixed forests dominated by plants of Fagaceae and Pinaceae. 

Distribution: Currently known from southwestern China and northwestern India.

Additional specimens examined: China, Yunnan Province: Baoshan, Mangkuan Town, Baihualing Village, 2100 masl, 7 August 2022, Mei-Zhi Tian 157 (KUN-HKAS128964); the same date and location, Mei-Zhi Tian 164 (KUN-HKAS128972) and Mei-Zhi Tian 173 (KUN-HKAS128965); Tengchong County, Tiantai Mountains, 2000 masl, 25 July 2022, Mei-Zhi Tian 051 (KUN-HKAS128963); Tengchong County, Zhonghe Town, Gaotian Village, 2040 masl, 13 August 2010, Yan-Jia Hao 252 (KUN-HKAS69236).

Notes: *Thelephora sikkimensis* was originally described from India and characterized by its imbricate to rosette-shaped basidiomes with branches arising from a shared stipe or center, pale orange to grayish green and non-zonate, warty hymenial surface, and rugose to warty and pale orange stipe [22]. 

*Thelephora aurantiotincta* has a warty and pale orange hymenial surface, subclavate to subcylindrical basidia, and irregularly lobed basidiospores. Such traits are very similar to *T. sikkimensis*, but *T. aurantiotincta* can be easily distinguished from *T. sikkimensis* by its non-zonate pale orange abhymenial surface [4,50]. The imbricate to rosette basidiomes, the zonate abhymenial surface, and the presence of cystidia of *T. ganbajun* make it similar to *T. sikkimensis*. However, *T. ganbajun* is different from *T. sikkimensis* by its gray to dark-brown abhymenial surface, gray to black hymenial surface, brown to yellowish brown stipe without warty ornamentation, and relatively short basidia (25–55 µm long in *T. ganbajun* vs. 72–100 µm long in *T. sikkimensis*). *Thelephora sikkimensis* is reported here for the first time from China.

Phylogenetically, *T. sikkimensis*, *T. petaloides*, and *T. lacunosa* clustered together with high support values. However, *T. petaloides* can be distinguished from *T. sikkimensis* by its big basidiomes (40–100 × 55–220 mm in *T. petaloides* vs. 45–67 × 50–70 mm in *T. sikkimensis*); nearly glabrous abhymenial surface which is brownish gray at the center becoming orange-yellow to grayish orange or pale orange elsewhere but chalky white at the margin; rugose and warty hymenium which is orange-gray at the center, bright orange elsewhere, and chalky white at the margin; rugose and glabrous brownish gray stipe; and a relatively thick context which is up to 4 mm thick. *Thelephora lacunosa* has medium-sized basidiomes (67–80 × 80–150 mm); lacunary, irregularly ridged, non-zonate, and warty abhymenial surface which is brown, grayish brown to grayish orange at the center, much paler towards the margin, and white to pale orange-gray at the margin; rugose and warty hymenium surface, brownish orange to gray or purple-gray at the center, pale orange towards the margin, and orange-white at the margin; and subconical stipe which is broadened or flatted at the base with a brownish red to brownish yellow surface.

### 3.4. Discussion

In this study, five species, *T. sikkimensis* new to China and *T. petaloides*, *T. lacunosa*, *T. dactyliophora*, and *T. pinnatifida* new to science, are reported based on a combination of phylogenetic analyses, genetic differences, and morphological features. In our phylogenetic analyses, *T. lacunosa*, *T. petaloides*, and *T. sikkimensis* clustered together with high support values, but the relationships among the three taxa are not well solved by the ML and Bayesian analyses. In addition, the diagnostic morphological characters among them are often subtle. It seems that these species might have started diverging from each other relatively recently. Genetic distance measurements have been used frequently as a tool to investigate species boundaries and to identify cryptic diversity in fungi [51,52,53]. Based on the Maximum Composite Likelihood model, the genetic variations between the three closely related species *T. lacunosa*/*T. sikkimensis*, *T. petaloides*/*T. sikkimensis*, and *T. lacunosa*/*T. petaloides* are 1.9–3%, 2.7–3.4%, and 2.1–3%, respectively, which are much higher than the within-species genetic variation (ranging from 0 to 0.9%). Morphologically, all of these five species have nodulose to verrucose ornamentations on the surface of basidiospores. But *T. sikkimensis*, *T. petaloides*, and *T. lacunosa* have imbricate basidiomes, while *T*. *dactyliophora* and *T. pinnatifida* have clavarioid basidiomes. It is evident that the shapes of basidiomes and the morphology of the hymenial surface are important characteristics distinguishing species of *Thelephora*. However, for the precise identification of *Thelephora* species, the size of basidiomes, basidia, and basidiospores; the shapes and size of the stipe; the color and zonation of abhymenial and hymenial surfaces; and the thickness of the context are also important characteristics. Detailed morphology comparisons among these five species are shown in Table 2. The combination of phylogenetic analyses, genetic differences, and morphological features can enhance the reliability and objectivity of species delimitation [54].

To date, 285 species of this genus have been reported and described (Index Fungorum: http://www.indexfungorum.org/Names/Names.asp, accessed on 6 April 2024), of which 29 (including the 5 species reported in this study) have been reported from China. China is one of the most biodiverse countries in the world. Like other groups of fungi, there are likely to be many *Thelephora* species that have not been scientifically described or remain to be discovered [55]. Further studies are needed to enrich the species diversity of this genus.

## Figures and Tables

**Table 1:** Species, vouchers, GenBank/UNITE accessions, and localities of specimens used in this study. Sequences newly generated in this study are shown in bold.

Taxa	Voucher	GenBank/UNITE No.	Locality	Reference
		ITS		
***Thelephora lacunosa***	**KUN-HKAS128966**	**OR512336**	**China**	**This study**
***T. lacunosa***	**KUN-HKAS128967**	**OR512337**	**China**	**This study**
***T. lacunosa***	**KUN-HKAS128971**	**OR512338**	**China**	**This study**
***T. lacunosa***	**KUN-HKAS128968**	**OR512335**	**China**	**This study**
*T. petaloides* (labeled as *T. sikkimensis*)	KUN-HKAS122715	ON794445	China	GenBank
***T. petaloides***	**KUN-HKAS128969**	**OR512332**	**China**	**This study**
***T. petaloides***	**KUN-HKAS128970**	**OR512333**	**China**	**This study**
***T. petaloides***	**KUN-HKAS97730**	**OR512334**	**China**	**This study**
***T. sikkimensis***	**KUN-HKAS128964**	**OR512329**	**China**	**This study**
***T. sikkimensis***	**KUN-HKAS128965**	**OR512331**	**China**	**This study**
***T. sikkimensis***	**KUN-HKAS128972**	**OR512330**	**China**	**This study**
***T. sikkimensis***	**KUN-HKAS128963**	**OR512328**	**China**	**This study**
*T. sikkimensis*	KD 16-042	MF684018	India	[22]
*T. wuliangshanensis*	CLZhao 3406	MZ400677	China	[14]
*T. wuliangshanensis*	CLZhao 3408	MZ400678	China	[14]
*T. vialis*	TENN-F-072094	MN121022	USA	[25]
*T. vialis*	TENN-F-072281H2	MN121029	USA	[25]
*T. pseudoganbajun*	YL3-10	KY245255	China	[25]
*T. pseudoganbajun*	Yuan16794	OP793768	China	[25]
*T. ganbajun*	KUN-HKAS14735	KY245240	China	GenBank
*T. ganbajun*	Yuan16715	OP793760	China	[25]
***T. dactyliophora***	**KUN-HKAS131941**	**OR940521**	**China**	**This study**
***T. dactyliophora***	**KUN-HKAS131943**	**OR940523**	**China**	**This study**
*T. pacifica*	VO2019_125	OR548196	Mexico	[40]
*T. pacifica*	VO2019_101	OR548195	Mexico	[40]
*T. penicillata*	X619	OL469899	Prague	GenBank
*T. penicillata*	X618	OL469898	Prague	GenBank
***T. pinnatifida***	**KUN-HKAS131946**	**OR940524**	**China**	**This study**
***T. pinnatifida***	**KUN-HKAS131947**	**OR940525**	**China**	**This study**
***T. pinnatifida***	**KUN-HKAS96412**	**OR940526**	**China**	**This study**
*T. iqbalii*	MH810	JX241471	Pakistan	[41]
*T. glaucoflora*	Dai13623A	OP793751	China	[25]
*T. glaucoflora*	Dai16612	OP793754	China	[25]
*T. grandinioides*	CLZhao 4107	MZ400675	China	[14]
*T. grandinioides*	CLZhao 21020	MZ400676	China	[14]
*T. nebula*	Yuan11516	OP793746	China	[25]
*T. nebula*	Yuan11515	OP793745	China	[25]
*T. austrosinensis*	GDGM48867	MF593265	China	[13]
*T. austrosinensis*	GDGM25680	MF593261	China	[13]
*T. aquila*	Wei8831	OP793743	China	[25]
*T. aquila*	Wei8833	OP793744	China	[25]
*T. palmata*	JMP0085	EU819443	USA	GenBank
*T. palmata*	iNAT:30911260	MZ276274	USA	GenBank
*T. palmata*	UBC: F33078	MF908479	Canada	GenBank
*T. palmata*	JLF3733	MK847520	USA	GenBank
*T. pseudoversatilis*	FCME26152	KJ462486	Mexico	[17]
*T. pseudoversatilis*	FCME26232	JX075890	Mexico	[17]
*T. versatilis*	MEXU27094	KC595628	Mexico	[17]
*T. versatilis*	FCME26141	NR154492	Mexico	[17]
*T. terrestris*	Hilszczanska D. 1-IBL	FJ532478	Poland	GenBank
*T. terrestris*	MT1-2203-3	EU427323	Finland	GenBank
*Odontia fibrosa*	SS38	MH310788	Sweden	[14]
*O. ferruginea*	UK18	UDB000285	Estonia	[14,18,22]

**Table 2:** Comparisons among species of *Thelephora* in this study.

Structures	*T. sikkimensis*	*T. petaloides*	*T. lacunosa*	*T. dactyliophora*	*T. pinnatifida*
Basidiome	Imbricate to rosette, small, 45–67 × 50–70 mm, with flabelliform to petaloid branches	Imbricate to umbrella, large, 40–100 × 55–220 mm, with flabelliform, applanate-lobate branches	Imbricate, rosette to flabelliform, medium-sized, 67–80 × 80–150 mm, with flabelliform, petaloid to applanate-lobate branches	Coralloid, small, 35–65 × 30–40 mm, with spathulate to narrow petaloid branches	Clavarioid, small, 35–45 × 28–85 mm, with clavate to pinnatifid or ramiform branches with tips needle-like
Abhymenium	Sulcate, indistinctly zonate, radially rugulose or wrinkled; grayish black at center, purplish gray to grayish green elsewhere, but chalky white to orange-white at margin	Sulcate to irregularly ridged, zonate, glabrous; brownish gray at center, orange-yellow to grayish orange or pale orange elsewhere, but chalky white at margin	Lacunal, sulcate, irregularly ridged, non-zonate, and warty; brown, grayish brown to grayish orange at center, much paler towards margin, but white to pale orange-gray at margin	Slightly rugulose, non-zonate; grayish yellow at center, brownish gray to gray, but chalky white to orange-white at margin	Without abhymenium
Hymenium	Imperceptibly rugose, warty, non-zonate, gradually from smooth to irregularly plicate near margin; pale orange to purplish gray or grayish green, but orange-white to almost white at margin	Imperceptibly rugose, warty, zonate near margin; orange-gray at center, bright orange elsewhere, but chalky white at margin	Imperceptibly rugose to warty, non-zonate; brownish orange to gray or purple-gray at center, pale orange towards margin, but orange-white at margin	Rugulose, non-zonate; brownish gray to gray, but chalky white to orange-white at margin	Predominantly amphigenous, sulcate, non-zonate; visibly ribbed; brownish orange to brown, but chalky white to orange-white at margin
Stipe	Irregularly cylindrical, 12–14 × 2–9 mm; surface rugose to warty, pale orange, sometimes purplish gray	Irregularly cylindrical to flatted or broadened at base, 10–40 × 9–13 mm; surface rugose, glabrous, brownish gray	Subconical to broadened or flatted towards base, 20–25 × 12–35 mm; surface rugose, glabrous, brownish red to brownish yellow	Irregularly cylindrical to flatted or broadened at base, 10–20 × 2–5 mm; surface smooth to slightly rugose, brownish gray	Irregularly cylindrical, 0–15 × 0–10 mm; surface rugose, brown
Context	1–2 mm thick	1–4 mm thick	0.5–3.5 mm thick	0.1–0.5 mm thick	0.1–2 mm thick
Basidia	72–100 × 7.5–10 µm	53–92 × 9–12 µm	72–100 × 8–12 µm	45–72 × 6–9 µm	47–90 × 10–11.5 µm
Basidiospore	4.5–6.5 × 4–5.5 µm	4.5–7.5 × 3.5–6.5 µm	4.5–6.5 × 4–5.5 µm	4–8.5 × 4–6.5 µm	5.5–9.5 × 5–8.5 µm

## Data Availability

Publicly available datasets were analyzed in this study (https://www.ncbi.nlm.nih.gov/, accessed on 6 April 2024; https://unite.ut.ee, accessed on 25 February 2024).

## Supplementary Materials

The following supporting information can be downloaded at https://www.mdpi.com/article/10.3390/jof10040300/s1: Table S1: Estimates of evolutionary divergence among sequences of *Thelephora*.
